# Effectiveness of data auditing as a tool to reinforce good research data management (RDM) practice: a Singapore study

**DOI:** 10.1186/s12910-021-00662-y

**Published:** 2021-07-28

**Authors:** Hui Xing Lau, Ser Lin Celine Lee, Yusuf Ali

**Affiliations:** Lee Kong Chian School of Medicine, Nanyang Technological University, Singapore, Singapore

**Keywords:** Data auditing, Research data management, Data management plan, Research integrity, Compliance

## Abstract

**Background:**

Institutions, funding agencies and publishers are placing increasing emphasis on good research data management (RDM). RDM lapses in medical science can result in questionable data and cause the public’s confidence in the scientific community to crumble. A fledgling medical school in a young university in Singapore has mandated every funded research project to have a data management plan (DMP). However, researchers’ adherence to their DMPs was unknown until the school embarked on routine data auditing. We hypothesize that research data auditing improves RDM awareness, compliance and reception in the school.

**Methods:**

We conducted surveys with research PIs and researchers before and after data auditing to evaluate differences in self-reported RDM awareness, compliance and reception. As it is mandatory to deposit research data in a central data repository system in the school, we tracked data deposition by each laboratory from 2 weeks before to 3 months after data auditing as a marker of actual RDM compliance.

**Results:**

Research data auditing had an overall positive effect on self-reported RDM awareness, compliance and reception for both research PIs and researchers. Research PIs agreed more that RDM was important to scientific reproducibility, were more aware of proper RDM, had higher RDM strength in their laboratories and were more compliant with the DMP. Both research PIs and researchers believed data auditing helped them to be more compliant with data deposition in the repository. However, data auditing had no significant impact on laboratories’ data deposition rates over time, which could be due to the short sampling period.

**Conclusions:**

Research PIs and researchers generally felt that data auditing was effective in improving RDM practices. It helped to evaluate their RDM practices objectively, propose corrective actions for RDM lapses and spread awareness of the university’s data management policies. Our findings corroborated other studies in medical research, geosciences, engineering and ethics that data auditing promotes good RDM practices. Hence, we recommend research institutions worldwide to adopt data auditing as a tool to reinforce research integrity.

**Supplementary Information:**

The online version contains supplementary material available at 10.1186/s12910-021-00662-y.

## Background

Good Research Data Management (RDM) is essential to scientific research, especially in this era of big data. RDM refers to the creation, processing, storage and sharing of research data. Data mismanagement was found to be one of the top three reasons for retraction of papers [1]. 583 of 2373 (24.6 %) retracted papers listed in the Retraction Watch database from 1 to 2018 to 29 August 2019 were also withdrawn due to poor handling of data (concerns/issues about, error in or unreliable data and image and non-reproducible results) [2]. Thus, it is important to manage research data properly to uphold research integrity and ensure reproducible results.

Many academic institutions, funding agencies and scientific publishers require projects to use a data management plan (DMP). A DMP documents how a researcher handles every step of the research data lifecycle and can be updated anytime. This encourages efficient research and smooth handover of projects and data among researchers. However, it is only beneficial if researchers understand its use and adhere to it throughout the project. Few academic institutions have implemented routine data auditing despite their potential in promoting adherence to good data management. A Data Audit Framework developed by University of Glasgow was tested in three pilot audits in University of Glasgow (geosciences), University of Edinburgh (geosciences) and University of Bath (mechanical engineering) and it helped researchers to acknowledge lapses in managing data and to become more knowledgeable in good RDM practices [3]. More studies are needed to ascertain the effectiveness of data auditing in upholding good RDM in different scientific fields and ensuring compliance with DMPs in order to aid in the formulation of research integrity policies.

A 9-year-old medical school in Nanyang Technological University Singapore (NTU) has implemented routine data auditing since July 2018. Although NTU requires all research principal investigators (PIs) to submit a DMP before research funds can be released, at the time of study, 17 out of 40 research PIs in the medical school had attended NTU’s DMP training workshops personally before data auditing commenced despite receiving several invitations to attend one. This suggested that good DMP writing was perhaps lower on their priority list compared to other tasks such grant writing, manuscript preparation and fulfilment of teaching duties. There were no checks to confirm that all researchers within the medical school complied with their projects’ DMPs. In addition, the medical school requires researchers to deposit all primary research data over the course of their projects in a dedicated central data repository system which serves to store data securely. The data repository is a physical drive with space allocated for data deposition by the medical school and is accessible using the intranet or virtual private network remotely. The objective of this data repository is to securely store all forms of research data, and this is not to be confused with a centralized institutional-level data repository system (DR-NTU) that serves to store and share published data (i.e. it provides a DOI to data files linked to published papers). It only had 29.5 % utilisation eight months after its 2018 inception based on the amount of used storage reported by the school’s IT department. There was a pressing need to improve researchers’ data management practices, hence data auditing of random laboratories was introduced. Through data auditing, research PIs and researchers should be more knowledgeable in proper RDM practices and non-compliances with RDM and DMP of a project should be identified.

The objective of this study was to determine whether there were changes to research PIs’ and researchers’ RDM awareness, compliance and reception and we hypothesise that data auditing results in significant improvements. To test the hypothesis, we conducted surveys with research PIs and researchers before and after data auditing to assess its impact on self-reported RDM awareness, compliance and reception. Secondly, we tracked the volume of data deposition into the data repository by each laboratory from 2 weeks before to 3 months after data auditing to determine whether it encouraged researchers to be more compliant. This study evaluated the effectiveness of data auditing in upholding good management of research data and will aid in formulating research integrity policies in research institutions worldwide.

## Methods

### Data auditing

Data auditing was performed by the school’s research integrity office. The auditor randomly chose a research project which was at least 6 months old to ensure that some research data had been generated. A pre-audit interview was conducted with the research PI of the laboratory to understand the project and to remind them to deposit data into the data repository. After 2 weeks, the audit commenced with the participation of researchers. The auditor checked if data files were named appropriately and uploaded into the data repository and provided immediate feedback to the researchers. After the audit, an audit report which highlighted aspects of RDM requiring improvements in the laboratory was sent to the research PI and it was left to his/her discretion whether to share it with the researchers. The auditor followed up with the laboratory to ensure that corrective actions were taken.

### Surveys of research PIs and researchers

A total of 25 research PIs and 31 researchers from 25 laboratories participated in pre- and post-audit surveys (Additional file 1). The protocol was approved by the Institutional Review Board in NTU Singapore (IRB-2019-09-029) and the study was pre-registered with the Open Science Framework (10.17605/OSF.IO/694E7). The surveys were Likert Scale questionnaires developed by the research team with 12 questions on self-reported (a) awareness of RDM (questions 1–6), (b) compliance with depositing data into the data repository (questions 7–9) and (c) reception to DMP (questions 10–12) (Table 1). Respondents rated on a scale from 1 to 10, with 10 being the most favourable reaction. The post-audit survey occurred four weeks after the pre-audit survey and the same set of questions were used.

**Table 1:** Questions used in pre and post-audit surveys

Question	
1	How important do you think is RDM in your research work?
2	How much do you agree with this statement: “Lack of reproducibility in science is because data is not properly managed”?
3	Rate your level of awareness of proper RDM
4	What do you think is the current strength of RDM in your laboratory?
5	How much of your time do you think you should devote to proper RDM?
6	Do you think more education and training is needed in RDM?
7	Rate how likely you will deposit ALL research data into the central data repository system
8	If deposition of research data into the central data repository system is not mandatory, rate how likely you will deposit research data into it
9	Rate your level of preference in having a service that helps to back up all your research data
10	Do you think your DMP will assist you in the proper storage and easy retrieval of data?
11	How useful do you think is DMP in reinforcing RDM?
12	Rate your current level of compliance with the DMP

### Data deposition of laboratories

We tracked data deposition in the data repository by each laboratory to evaluate if data auditing increased the researchers’ compliance. The sampling points were 2 weeks before an audit (0 week), start of audit (2 weeks), end of audit (4 weeks), 1 month post-audit (8 weeks) and 3 months post-audit (16 weeks), forming four sampling intervals. We also recorded the data deposition of five controls, which were randomly-selected non-audited laboratories that were using the data repository. While 16 audited laboratories were tracked, we only analysed those which had been using the data repository before data auditing commenced and did not remove data from the data repository (five laboratories) in order to match the controls.

### Data analysis of surveys

For the surveys, the research PIs and researchers were analysed separately. If a respondent provided multiple responses to a question and the answers differed by one, the mean was taken. Otherwise, the affected question was excluded from the analysis for both pre- and post-audit surveys. Statistical analyses were performed. We used the Shapiro-Wilk test to check the normality of the answers and boxplots to check whether the distribution was symmetrical (IBM SPSS Statistics). We then performed sign tests to determine if there was a significant change in the answers for each question before and after the audit (α = 0.05) (IBM SPSS Statistics). The test disregarded ties in answers. 95 % confidence interval of the difference in medians for responses which were not ties was calculated (GraphPad Prism).

The difference in total scores of each respondent between pre- and post-audit surveys was analysed to determine whether there was an overall improvement in RDM awareness, compliance and reception after the audit. If some questions were excluded due to multiple answers, the total score was calculated by multiplying the mean of the valid answers by 12. We used the Shapiro-Wilk test to check the normality of the total scores and subsequently carried out paired *t* tests on the scores of research PIs and researchers separately (α = 0.05) (IBM SPSS Statistics).

### Data analysis of data deposition of laboratories

The rates of data deposition by the audited laboratories were compared to the deposition by controls over four sampling intervals to determine if data auditing encouraged researchers to be more compliant. Data deposition rate of a sampling interval was calculated using $Rate=\frac{{Data}_{2}-{Data}_{1}}{Number of weeks}$, where Data_1_ and Data_2_ represent data deposition at the earlier and later sampling points respectively. The Shapiro-Wilk test was carried out to check the normality of the rates (IBM SPSS Statistics). We then used the F1-LD-F1 design from nparLD package to perform non-parametric longitudinal analysis (α = 0.05) [4]. The between-subjects factor was group (audited laboratories and controls) and the within-subject factor was time (four sampling intervals). A significant main group effect where audited laboratories had higher data deposition rates than controls would support our hypothesis.

## Results

### Surveys for research PIs

We received a 100 % response rate for the surveys. We observed significant differences between the pre- and post-audit answers for questions 2, 3, 4, 7 and 12 (Table 2). No significant differences were found for other questions.

**Table 2:** Results for sign test comparing pre- and post-audit answers from research PIs

Q	Differences			*p*	95% CI		
	+	−	Tie		Lower	Upper	Actual CI (%)
1	8	5	12 (11)	0.581	− 1.00	2.00	97.8
2	14	4	7 (3)	**0.031**	1.00	2.00	96.9
3	15	5	5 (1)	**0.041**	1.00	2.00	95.9
4	18	5	2 (0)	**0.011**	1.00	2.00	96.5
5	9	5	11 (6)	0.424	− 2.00	2.00	98.7
6	12	5	8 (3)	0.143	− 1.00	2.00	95.1
7	12	1	12 (10)	**0.003**	1.00	3.00	97.8
8	12	5	8 (7)	0.143	− 1.00	1.00	95.1
9	6	4	15 (12)	0.754	− 1.00	2.00	97.9
10	12	9	4 (0)	0.664	− 1.00	2.00	97.3
11	11	8	6 (0)	0.648	− 1.00	2.00	98.1
12	12	2	11 (2)	**0.013**	1.00	2.00	98.7

Values in boldface denote significant differences (*p* < 0.05). Numbers in parentheses represent number of answers which were 10 in both the pre-audit and post-audit surveys. n = 25

We compared the total scores of the surveys to check if data auditing had a significant impact on RDM in general. The total scores for research PIs showed a significant difference after data auditing (*p* = 0.003, 95 % CI [2.481, 10.639]).

### Surveys for researchers

Likewise, for researchers, we performed the sign test that showed a significant difference between pre- and post-audit answers for question 7 (Table 3). No significant differences were found for other questions.

**Table 3:** Results for sign test comparing pre- and post-audit answers from researchers

Q	Differences			*p*	95% CI		
	+	−	Tie		Lower	Upper	Actual CI (%)
1	6	7	18 (15)	1.000	− 2.00	1.00	97.8
2	14	5	12 (2)	0.064	− 1.00	2.00	98.1
3	13	5	12 (4)	0.096	− 1.00	2.00	96.9
4	13	7	11 (3)	0.263	− 1.00	1.00	95.9
5	10	11	10 (6)	1.000	− 1.00	1.00	97.3
6	8	14	9 (4)	0.286	− 2.00	1.00	98.3
7	15	4	11 (8)	**0.019**	1.00	2.00	98.1
8	12	5	14 (8)	0.143	− 1.00	2.00	95.1
9	7	8	16 (14)	1.000	− 1.00	1.00	96.5
10	9	5	17 (8)	0.424	− 2.00	1.00	98.7
11	12	7	12 (5)	0.359	− 1.00	1.00	98.1
12	12	5	14 (5)	0.143	− 1.00	2.00	95.1

Value in boldface denotes significant difference (*p* < 0.05). Numbers in parentheses represent number of answers which were 10 in both the pre-audit and post-audit surveys. n ≥ 30

A significant difference was observed for the total scores of researchers (*p* = 0.021, 95 % CI [0.546, 6.093]).

### Data deposition of laboratories

Data depositions into the data repository for five audited laboratories and five controls were tracked to determine if data auditing encouraged laboratories to be more compliant. We calculated the rate of data deposition for each laboratory and performed a non-parametric longitudinal analysis using nparLD F1-LD-F1 design to determine if there were significant group, time and interaction effects (Table 4). The main group effect was not significant (*p* = 0.158), hence the data deposition rates were not significantly different between audited laboratories and controls. The main time effect was not significant as well (*p* = 0.642), which suggests that there were no significant changes in data deposition rates over time for both groups. Lastly, no significant interaction between group and time was observed (*p* = 0.256).

**Table 4:** Results of F1-LD-F1 nparLD

	*df*	*F*	*p*
Group	1	1.992	0.158
Time	1.873	0.423	0.642
Group x time	1.873	1.364	0.256

The independent factors were group (audited vs. controls) and time (4 sampling intervals). *df* = degrees of freedom, *F* = F value and *p* = *p *value. α = 0.05

**Fig. 1:**
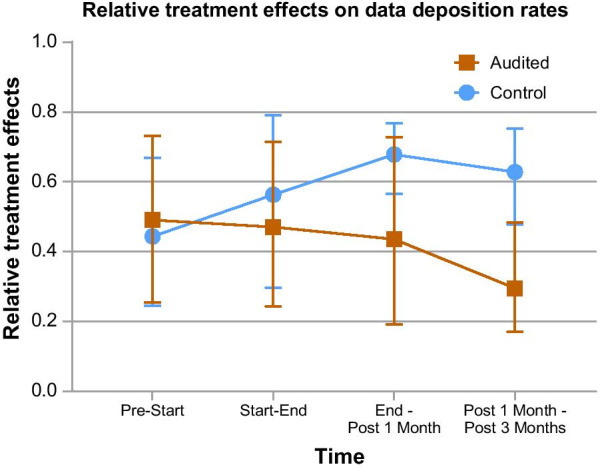
Relative treatment effect of data deposition rates over different time periods. Each point represents the relative treatment effect (audit laboratories, n = 5 or controls, n = 5) on data deposition rate per week for each time interval over the study period of 4 months. Error bars represent 95 % confidence interval.

The nparLD package determines if samples originate from the same distribution by deriving relative treatment effects from mean ranks. From the graph of relative treatment effects (Fig. 1), it is observed that audited laboratories generally had lower data deposition rates over time as compared to controls. For the period from before to start of data auditing, audited laboratories had slightly higher but insignificant data deposition rates than controls. The insignificant difference was also supported by the insignificant interaction effect.

## Discussion

This study aimed to uncover if data auditing was effective in improving RDM awareness, compliance and reception, particularly on data creation, processing, storage, which would help to reinforce research integrity in NTU and other institutions worldwide. Given this scope, we did not include the data sharing aspect of RDM. The latter is critical for research data validation and reuse but itself demands a separate detailed study. There were significant improvements in agreement that improper data management caused lack of reproducibility in science (question 2), awareness of proper RDM practices (question 3), strength of RDM in the laboratories (question 4) and compliance with DMP (question 12) for research PIs and likelihood of depositing all data into the data repository (question 7) for both research PIs and researchers after data auditing. Data auditing had an overall positive impact on both research PIs and researchers. However, data deposition rates of the laboratories were not significantly affected by data auditing.

For research PIs, data auditing highlighted how RDM lapses can impact future use and retrievability of data, thus they believed more strongly in the importance of good RDM. The one-to-one evaluation of RDM practices in their laboratories could be effective in improving their RDM awareness and practices. The importance of the data repository was emphasised during data auditing, hence research PIs were more inclined to follow the school’s policy. Previously, research PIs might view DMP as a means to open a research grant account. Data auditing allowed them to realise that the school placed a high importance on compliance with DMP.

No significant differences were found for other questions. Research PIs might had already felt that RDM was important in research work. They might had also felt that current efforts were sufficient in maintaining good RDM, which limited their view that additional data auditing may help in improving RDM practices. They were probably used to their own system of depositing data before joining the school and poor adoption rates could be linked to concerns about data security. Many research PIs already preferred having a service to back up data, hence data auditing had no significant impact on their preferences. Despite being more compliant with their DMPs, they might had felt that they were the ones implementing good RDM practices in the laboratory while the DMP was simply regarded as a document that recorded these practices. Thus, they did not feel that the DMP was necessary in managing research data. Data auditing helped to evaluate their research laboratories’ RDM practices objectively, propose corrective actions for RDM lapses and spread awareness of the university’s data management policies. Thus, research PIs generally found it to be effective in promoting good RDM.

Researchers felt they were more likely to deposit all research data into the data repository. Research PIs usually delegate the task of depositing data to researchers. In addition, researchers were more involved in the compliance check which mostly examined data in the data repository. Thus, researchers realised that depositing data in the data repository was mandatory and were more willing to follow the school’s policy.

No significant differences were found for other questions. Similar to research PIs, researchers might had already felt that RDM was important in research work. They might also had felt that other factors, such as robustness of experimental design, contributed more to reproducibility than improper data management. Although they were heavily involved in the compliance check, researchers might not have received the formal data audit report that contained audit findings that included detailed explanations on the current RDM strength and corrective actions for RDM lapses in their respective laboratories. Consequently, their perception of RDM did not change significantly. Researchers might prefer to store data in devices such as external hard drives where they can edit and sort data freely as opposed to the data repository where they were unable to delete these files. Most researchers already preferred having an automatic back-up service for their data, thus no significant improvement was observed after data auditing. Researchers’ data management practices were likely a result of the research PI’s instructions since they did not write or update DMPs. Hence, they did not find DMPs useful and were not more compliant.

Even though data auditing did not cause significant improvements in specific areas such as the perceived usefulness of DMP, it did result in an overall positive impact on researchers. In addition to instructions from research PIs and training courses, it provided another avenue for researchers to learn more about proper RDM practices. If they were more deeply involved in planning data management, such as the writing of DMPs, they might had appreciated the usefulness of data auditing in upholding good RDM practices more.

While the survey results suggested that laboratories were more compliant with good RDM practices after data auditing, data deposition into the data repository was not significantly affected. The insignificant group effect shows that audited laboratories did not utilise the data repository more or less than controls. The time effect was not significant as well, which implies that audited laboratories and controls maintained a steady rate of deposition over the sampling period. The interaction between group and time effects was also insignificant. This may mean that the research PIs and researchers felt that data auditing was important and were willing to deposit data into the data repository, but they either did not persist in complying or did not generate much data in the 16 weeks sampling period after data auditing ended.

Our findings provided more evidence that auditing is effective in promoting awareness, compliance and reception with regard to good research practices. They corroborated University of Glasgow, University of Edinburgh and University of Bath’s conclusions that data auditing was beneficial to good RDM [3]. Similar to our study, these institutions evaluated a data auditing framework where auditors identified and understood more about potential research projects to be audited, assessed research data files and management, interviewed researchers on data management practices and generated an audit report to share good RDM practices with researchers. Our results also supported other studies on effectiveness of data auditing in medical research. Clinical trials funded by the National Cancer Institute are regularly audited and researchers exhibited improved compliance with protocols, had deeper knowledge of RDM and had higher data quality [5, 6]. Routine data auditing of clinical quality registries helped to improve data quality as well [7, 8]. Moreover, Institutional Review Boards and Institutional Animal Care and Use Committees, established to safeguard the welfare of human and animal subjects in research studies respectively, conduct routine auditing of approved protocols which were found to proactively detect non-compliances and facilitate discussions between the ethics committee and the research team [9]. Since auditing in ethics is commonplace to protect human and animal subjects, auditing should also be incorporated into data management to protect research integrity given its effectiveness.

Data auditing can complement existing methods in upholding research integrity. Currently, research integrity is enforced by rules, education and whistleblowing [10]. However, the Open Science Collaboration (2015) found that only 39 % of behavioural studies were reproducible [11], which indicates that current measures were insufficient. Data auditing helps to determine the degree of correspondence between published and original source data, ensuring that researchers produce results that are reproducible, accurate and accountable [10]. It was estimated that 7 % of researchers are engaged in questionable research practices due to carelessness or fraud and a data audit can potentially cut down such incidence by half, therefore reinforcing research integrity [12]. Moreover, in reviewing Office of Research Integrity (ORI) misconduct files, it was found that three quarters of mentors (faculty members) had not reviewed the source data of their trainees and two-thirds had not set Responsible Conduct of Research standards for them [13]. Therefore, data auditing serves as a reminder for researchers to practise good RDM practices and helps to ensure data accountability, reproducibility and research integrity.

To complement the effectiveness of data audits, the following measures can be implemented to reinforce compliance of researchers with proper RDM practices. First, the PI of each research group can assign a senior research staff to be the RDM manager to help train new research hires on proper data storage in the school data repository and data documentation from the first day of work, before the commencement of any research work. Data Stewards assigned to each faculty has been suggested prior to this [14]. Second, it can be made mandatory for new research hires to undertake a compulsory e-learning course on proper RDM practices as part of their onboarding process with a minimal passing rate for a compulsory quiz. PIs can also make it mandatory for research staff to attend the DMP training/writing workshops so that they will have better understanding of the data lifecycle and data management process. Each researcher should also be cognizant of the DMP for each research project before embarking on any research data collection. Lastly, as highlighted in our study, it will be a good practice for the PIs to share outcomes of the data audits (i.e. RDM lapses documented in the audit reports) with their research groups during their laboratory meetings so that research staff are kept updated on their RDM strengths, weaknesses as well as on any other follow-up corrective actions required to rectify non-compliances.

## Limitations

In this study, we focused on proper storage of research data in RDM especially when tracking actual RDM compliance. However, RDM also encompasses sharing of data for validation and reuse. This aspect of RDM was only covered briefly in DMPs where research PIs and researchers had to outline their data sharing plans, but this was not included in our study design. Due to the pre- and post-survey design, the surveys were not anonymous. This might had led to social desirability bias where research PIs and researchers might not had answered truthfully and gave high ratings in order to maintain a good image. As the study was only conducted in the medical school, we were also restricted by small sample sizes. Moreover, although the five controls were chosen randomly, four happened to have very high data deposition rates or were consistently producing large amounts of data. Thus, data auditing might not appear to have significantly improved compliance with depositing data into the data repository when compared to controls. Tracking of data deposition was only performed for 16 weeks, hence we were unable to evaluate actual RDM compliance over a longer period of time. This study also assumed that laboratories produced data consistently within the 16 weeks period, when in reality data production could be irregular. Lastly, we were only able to evaluate the effectiveness of data auditing over one audit cycle. The medical school audits a laboratory every 1.5 years. A review has shown that repeated data auditing reduces data errors by 50% [15], hence evaluating data auditing over several cycles may show further improvement in awareness, compliance and reception to RDM.

## Conclusions

Data auditing, at its inception, generally improved RDM awareness, compliance and reception for both research PIs and researchers in the medical school. Research PIs reacted more favourably to data auditing where they felt it improved awareness of importance of RDM and proper RDM practices, RDM strength in their laboratories, compliance with depositing data into the data repository and compliance with DMP. Researchers felt that they were more compliant with data deposition into the data repository. There were no significant declines after data auditing. Data auditing did not affect data deposition rates significantly, which could be a consequence of small sample sizes and data production patterns. Overall, we believe that routine data auditing has good potential in reinforcing research integrity and can be adopted by other medical institutions. For future research, we can evaluate the effectiveness of data auditing on a larger scientific research community and explore other aspects of compliance, such as developing metrics for RDM. It would be useful to disseminate findings from this study to research integrity policymakers and researchers to increase recognition of data audit as a tool to promote good RDM. A data audit framework, similar to one developed by Jones and Ball et al. [3], can be created to guide other research institutions on implementing data auditing.

## Supplementary Information

**Additional file 1.** Pre- and post-audit survey questions on Research Data Management practices.

## Data Availability

The datasets supporting the conclusions of this article are available in the DR-NTU repository, 10.21979/N9/PXZSCB.

## Abbreviations

DMP: Data management plan
NTU: Nanyang Technological University Singapore
PI: Principal investigator
RDM: Research data management
