# Supplementary material for: Effectiveness of data auditing as a tool to reinforce good research data management (RDM) practice: a Singapore study
Source: BMC Med Ethics. 2021 Jul 28;22:103. doi: 10.1186/s12910-021-00662-y (PMC8317325; doi:10.1186/s12910-021-00662-y)
Supplement: Supplementary file 1 — Additional file 1. Pre- and post-audit survey questions on Research Data Management practices. [file 12910_2021_662_MOESM1_ESM.pdf]

Survey on Research Data Management (RDM) practices at LKCMedicine

Pre-audit survey

Prepared by LKCMedicine GRPO

DMP audit#:

Project:

1. How important do you think is RDM in your research work?

Unimportant ☐☐☐☐☐☐☐☐☐☐ Very Important
1 2 3 4 5 6 7 8 9 10

2. How much do you agree with this statement:

“Lack of reproducibility in science is because data is not properly managed”

Disagree ☐☐☐☐☐☐☐☐☐☐ Agree
1 2 3 4 5 6 7 8 9 10

3. Rate your level of awareness of proper RDM

Hardly aware ☐☐☐☐☐☐☐☐☐☐ Highly aware
1 2 3 4 5 6 7 8 9 10

4. What do you think is the current strength of RDM in your laboratory

Very weak ☐☐☐☐☐☐☐☐☐☐ Very strong
1 2 3 4 5 6 7 8 9 10

5. How much of your time do you think you should devote to proper RDM?

Hardly any time ☐☐☐☐☐☐☐☐☐☐ As much time as necessary
1 2 3 4 5 6 7 8 9 10

6. Do you think more education and training is needed in RDM?

It is enough ☐☐☐☐☐☐☐☐☐☐ Much is needed

1 2 3 4 5 6 7 8 9 10

7. Rate how likely you will store ALL research data on SDS

Very unlikely ☐☐☐☐☐☐☐☐☐☐ Very likely

1 2 3 4 5 6 7 8 9 10

8. If storage of research data on SDS is not mandatory, rate how likely you will store research data ***within*** SDS.

Very unlikely ☐☐☐☐☐☐☐☐☐☐ Very likely

1 2 3 4 5 6 7 8 9 10

9. Rate your level of preference in having a service that helps to back up all your research data

Very reluctant ☐☐☐☐☐☐☐☐☐☐ Highly preferred

1 2 3 4 5 6 7 8 9 10

10. Do you think your DMP will assist you in the proper storage and easy retrieval of data?

Hardly useful ☐☐☐☐☐☐☐☐☐☐ Very useful

1 2 3 4 5 6 7 8 9 10

11. How useful do you think is DMP in reinforcing RDM?

Hardly useful ☐☐☐☐☐☐☐☐☐☐ Very useful

1 2 3 4 5 6 7 8 9 10

12. Rate your current level of compliance to DMP

Hardly compliant ☐☐☐☐☐☐☐☐☐☐ Very compliant

1 2 3 4 5 6 7 8 9 10

Pre-audit survey acknowledged by:

PI/Researcher Name/ Date /Signature:

GRPO Name/ Date/ Signature:

Survey on Research Data Management (RDM) practices at LKCMedicine

Post-audit survey

Prepared by LKCMedicine GRPO

DMP audit#:

Project:

1. How important do you think is RDM in your research work?

Unimportant ☐ ☐ ☐ ☐ ☐ ☐ ☐ ☐ ☐ ☐ Very Important
1 2 3 4 5 6 7 8 9 10

2. How much do you agree with this statement:

"Lack of reproducibility in science is because data is not properly managed"

Disagree ☐ ☐ ☐ ☐ ☐ ☐ ☐ ☐ ☐ ☐ Agree
1 2 3 4 5 6 7 8 9 10

3. Rate your level of awareness of proper RDM

Hardly aware ☐ ☐ ☐ ☐ ☐ ☐ ☐ ☐ ☐ ☐ Highly aware
1 2 3 4 5 6 7 8 9 10

4. What do you think is the current strength of RDM in your laboratory

Very weak ☐ ☐ ☐ ☐ ☐ ☐ ☐ ☐ ☐ ☐ Very strong
1 2 3 4 5 6 7 8 9 10

5. How much of your time do you think you should devote to proper RDM?

Hardly any time ☐ ☐ ☐ ☐ ☐ ☐ ☐ ☐ ☐ ☐ As much time as necessary
1 2 3 4 5 6 7 8 9 10

6. Do you think more education and training is needed in RDM?

It is enough ☐☐☐☐☐☐☐☐☐☐ Much is needed

1 2 3 4 5 6 7 8 9 10

7. Rate how likely you will store ALL research data on SDS

Very unlikely ☐☐☐☐☐☐☐☐☐☐ Very likely

1 2 3 4 5 6 7 8 9 10

8. If storage of research data on SDS is not mandatory, rate how likely you will store research data ***within*** SDS.

Very unlikely ☐☐☐☐☐☐☐☐☐☐ Very likely

1 2 3 4 5 6 7 8 9 10

9. Rate your level of preference in having a service that helps to back up all your research data

Very reluctant ☐☐☐☐☐☐☐☐☐☐ Highly preferred

1 2 3 4 5 6 7 8 9 10

10. Do you think your DMP will assist you in the proper storage and easy retrieval of data?

Hardly useful ☐☐☐☐☐☐☐☐☐☐ Very useful

1 2 3 4 5 6 7 8 9 10

11. How useful do you think is DMP in reinforcing RDM?

Hardly useful ☐☐☐☐☐☐☐☐☐☐ Very useful

1 2 3 4 5 6 7 8 9 10

12. Rate your current level of compliance to DMP

Hardly compliant ☐☐☐☐☐☐☐☐☐☐ Very compliant

1 2 3 4 5 6 7 8 9 10

Post-audit survey acknowledged by:

PI/Researcher Name/ Date/Signature:

GRPO Name/ Date/ Signature:
